# Reforming Cancer Multidisciplinary Team Meetings: Introducing a Novel Clinical Radiological Assessment Meeting (CRAM) to Reduce Response Times and Workloads

**DOI:** 10.7759/cureus.79140

**Published:** 2025-02-17

**Authors:** Ayaz Ahmed Memon, Chintamani Godbole, Alexios Tzivanakis, Faheez Mohamed, Sanjeev Dayal, BJ Moran, Tom Cecil

**Affiliations:** 1 Peritoneal Malignancy Institute, Basingstoke and North Hampshire Hospital NHS Trust, Basingstoke, GBR

**Keywords:** colorectal cancer, gi radiology, mdt, multidisciplinary decision-making, peritoneal malignancy

## Abstract

Introduction

Multidisciplinary team (MDT) meetings are now considered part of the standard of care for decision-making and management of patients with cancer. However, most MDTs now face capacity issues and supplementary approaches should be considered. We report our experience with a novel clinical radiological assessment meeting (CRAM) as a 'mini-MDT' to expedite decision-making and enhance the function of the parallel specialist MDT.

Methods

A retrospective analysis of new referrals to a high-volume peritoneal malignancy unit between September 2016 and August 2018 was performed. Time to first response and decision following referral were assessed for the traditional referral pathway and after the introduction of the CRAM in September 2017. Response times were calculated from the receipt of the referral to the date of the first response and were classified into one of four categories: 'specialist peritoneal malignancy MDT review,' 'outpatient review,' 'recommendation for local follow-up', or 'further information required'. The Mann-Whitney U test was used to compare the response times between the two pathways.

Results

In total, 1478 new referrals were received in the two-year period, 769 pre-CRAM and 709 after CRAM introduction. The median referral to first response time was eight days using traditional pathways and five days after the introduction of the CRAM (p <0.001). In the traditional pathway, 234/769 (30.4%) patients were discussed further in the specialist MDT, compared with 122/709 (17.2%) after the CRAM assessment.

Conclusion

A novel CRAM significantly reduced first response times to the referring team facilitating rapid and safe assessment with quicker decisions for the patients. It enabled more appropriate use of an ever-expanding MDT.

## Introduction

A multi-disciplinary team (MDT) approach is now the accepted standard of care for cancer management, aiming to optimize treatment and improve cancer outcomes by coordinating expertise between different specialities [1, 2]. Many healthcare systems globally have made MDT discussion mandatory and linked MDT management to unit accreditation and remuneration [3, 4].

Whilst a laudable approach, there are some challenges and downsides to MDT decision-making such as such as unclear responsibility for decision-making, optimizing discussions, and maintaining focus in a large group. Cost and capacity are also an issue; with a large group of staff involved in MDT meetings, overruns either impact decision-making or other hospital activities and lack of capacity can delay treatment decisions. These issues are exacerbated in patients with metastatic disease where several MDTs may need to be involved. Indeed, for colorectal cancer patients with metastatic disease to the liver, lung, or peritoneum, MDT discussion may include the primary colorectal cancer MDT, and a combination of hepatobiliary, thoracic surgery, and peritoneal malignancy MDTs respectively.

Recently NHS England guidance has suggested MDT streamlining be considered as a strategy to improve its efficiency. This aims to ensure that adequate time gets allocated for discussion of the complex cases, by creating more flexibility in the management of MDTs. The method suggested is to divide the referred cases into two groups, those cases that need full discussion due to their complexity and those that can be deliberated upon by following standard-of-care protocols, without detailed discussion. This strategy can ensure a judicious use of the limited MDT time while facing a large volume of referrals [5].

Peritoneal malignancy is increasingly detected at baseline or during follow-up radiological imaging after primary cancer treatment, and there is increasing awareness of the role of cytoreductive surgery (CRS) and hyperthermic intraperitoneal chemotherapy (HIPEC), in selected patients with resectable peritoneal metastases [6-8]. Referrals emanate from colorectal, gynaecological oncology, upper gastro-intestinal, hepato-pancreatic-biliary and medical oncology specialist teams and patients will have been discussed in one, or more MDTs prior to referral. Frequently, a rapid evaluation, and opinion, are needed regarding the suitability of a patient for CRS and HIPEC.

The Peritoneal Malignancy Institute (PMI) in Basingstoke was established in 2000 as the first UK and Ireland peritoneal malignancy centre. PMI Basingstoke is one of the highest-volume centres globally, currently performing over 400 CRS and HIPEC cases annually with numbers increasing annually [9]. Traditionally, new referrals were allocated to the different PMI Consultants with little coordination and, when necessary, further information and a review of scans were requested to provide an opinion. This process was often administratively burdensome with potential for delays and commonly the specialist MDT (SMDT) became a forum for reviewing imaging rather than decision-making.

The Clinical Radiology Assessment Meeting (CRAM) was established to streamline case processing and provide reliable, timely opinions in response to a steadily increasing volume of new referrals and to facilitate the selection of cases requiring input from the peritoneal malignancy specialist MDT.

This article was previously presented as a meeting abstract (poster presentation) at the 41st Congress of the European Society of Surgical Oncology (ESSO) on October 19, 2022.

## Materials and methods

This is a retrospective study of a prospectively maintained database of all peritoneal malignancy referrals from September 2016 to August 2018. The referral process involves full clinical, surgical, and pathological details combined with electronic transfer of the patients' cross-sectional imaging. Two cohorts of patients were identified, those processed through the traditional referral pathway (September 2016 - August 2017) and those managed through the CRAM meeting September 2017 - August 2018). The Basingstoke Hospital Ethical Committee exempted this study from ethical approval as it was a service evaluation.

Traditional referral pathways

Traditionally, new referrals were distributed daily amongst the PMI surgical consultants with the main initial referral response being a letter of acknowledgement to the referrer with a plan to ask the PMI radiologists for a specialist review of scans. After the radiology report was concluded, options then included either SMDT review, outpatient review, further communication with the local teams, or the patient being referred back for local management if not considered suitable for treatment. 

CRAM pathway

CRAMs were introduced at our institution in September 2017 to achieve a methodical and timely processing of new referrals. The CRAM was scheduled weekly and included one of the surgeons specializing in peritoneal malignancy, a specialist radiologist, a clinical nurse specialist, and a member of the administration team. A pre-designed proforma was used to ensure that all the information and scans were available for discussion. Following discussion in the CRAM meeting, a letter was dictated by the team summarizing the clinical situation and outlining a plan. If the referring clinicians were seeking advice on follow-up and monitoring, the appropriate proformas were sent to the referring team and it was common to offer a review of histopathology slides, if not already performed. For those patients considered potentially suitable for CRS and HIPEC, an outpatient review was arranged. For complex decisions, the patient may at this stage be rediscussed at the SMDT. If patients were clearly unsuitable for surgery the decision was sent back to the referring team. The administrative team completed the response letters on the day of the CRAM for a rapid turnaround.

Specialist MDT (SMDT)

PMI Basingstoke SMDTs include peritoneal malignancy surgeons, specialist radiologists, clinical nurse specialists, pathologists, a clinical psychologist, SMDT co-ordinators and department managers. The SMDT commenced with a pathological review of CRS resection specimens to determine follow-up and treatment options. The subsequent section focused on new referrals and discussion of patients with updated scans and patients with recurrent disease after prior CRS. Finally, the SMDT concludes with a clinical, radiological and psychological assessment of operative cases planned for surgery in the following week. 

We report the impact of the CRAM meeting on new referral processing, referral to first response times and the decision made (review in SMDT, outpatient review or local follow-up). The referral to response time was defined as the time period from the date of receipt of the referral to the decision-making date when an initial treatment plan for the patient was outlined. The referral to first response times were calculated from the department referral database.

Continuous variables were represented as median values with an interquartile range. Categorical variables were represented as values and percentages. Referral to response times was compared between the two groups using the Mann-Whitney U test as our data set did not follow a normal distribution. 

## Results

**Figure 1:**
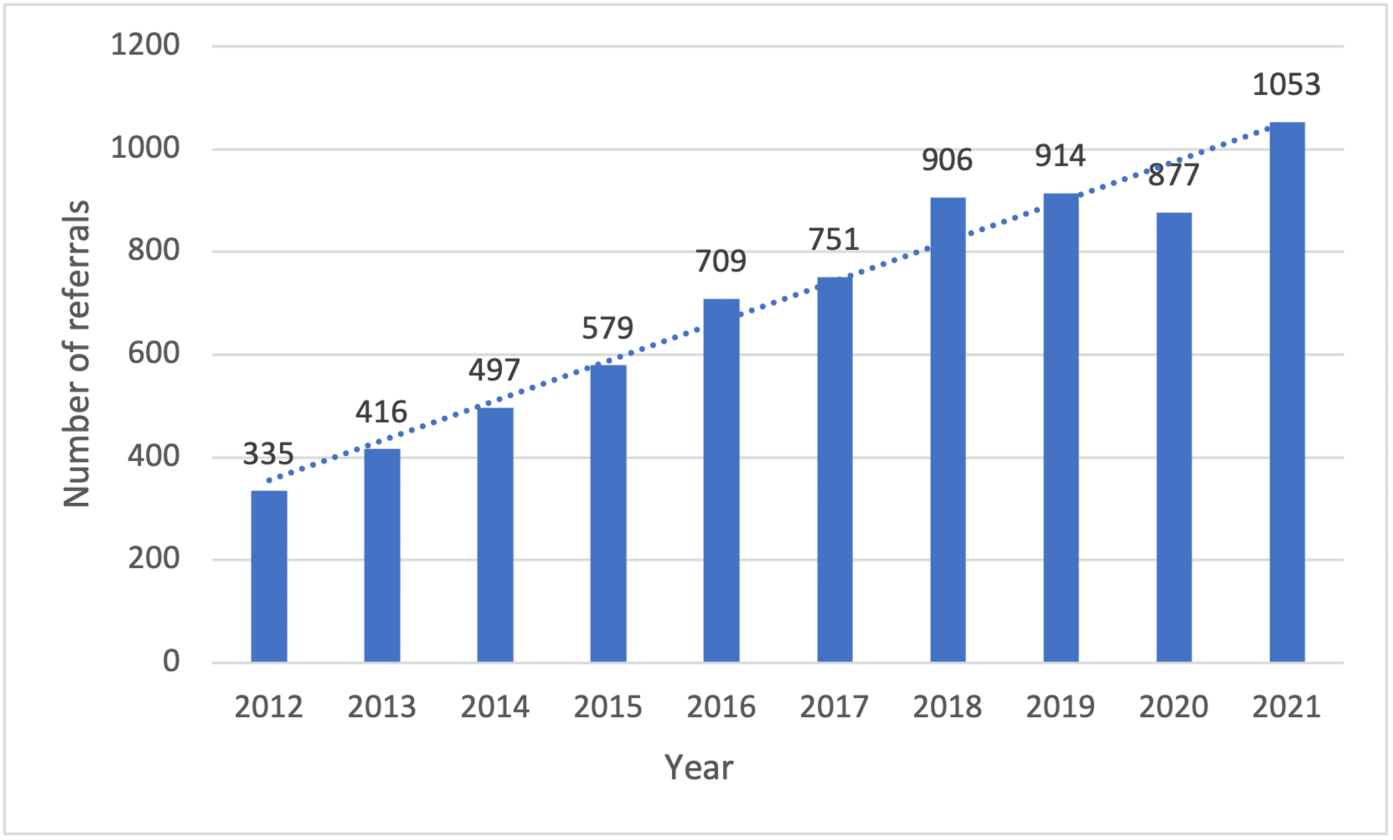
New referrals per year to the Peritoneal Malignancy Institute

**Figure 2:**
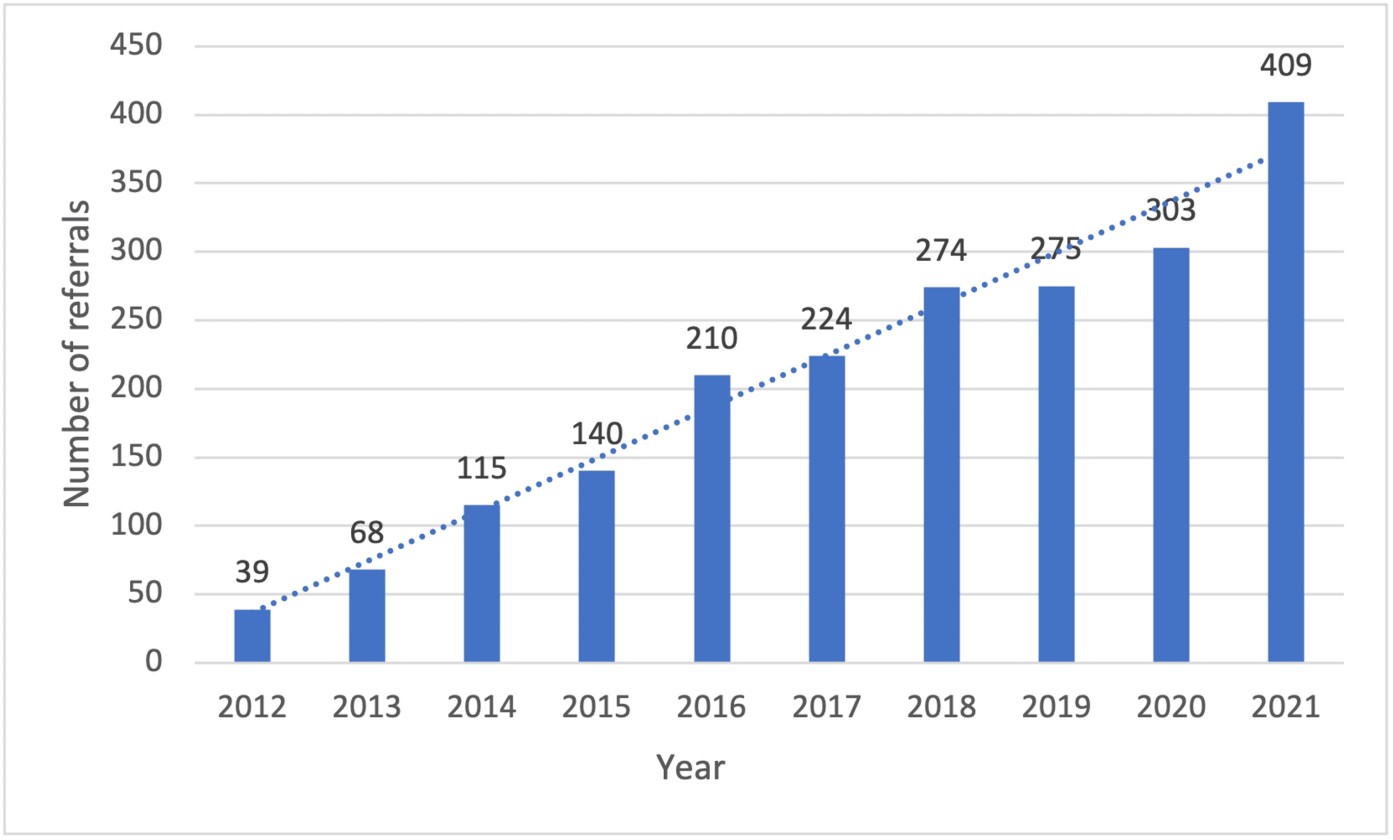
New referrals per year with colorectal peritoneal metastases

The number of new referrals per year to the Peritoneal Malignancy Institute and referrals with colorectal peritoneal metastases continue to increase annually (Figures 1-2). A total of 1478 new peritoneal malignancy patient referrals were received over the two-year study period. A total of 769 patients were referred using the traditional referral pathway (September 2016 - August 2017) and 709 referrals were processed in the CRAM pathway (September 2017 - August 2018). 

Table 1 outlines decisions made at first response using both pathways. A total of 30.4% (n=234) of the patients were discussed in the SMDT in the traditional pathway. The introduction of CRAM resulted in the cases being referred to SMDT reduced to 17.2% (n=122). The proportion of the patients who were referred for outpatient review remained nearly the same in both traditional and CRAM pathways being 27.4% (n=211) and 29.3% (n=208) respectively. With the introduction of CRAM, the number of patients referred back for local follow increased from 40.7% (n=313) to 53.4% (n=379). With the introduction of CRAM, no patient had a deferred response due to lack of information, while this was 1.4% (n=11) in the traditional pathway.

**Table 1:** Decision at first response CRAM: clinical radiological assessment meeting; SMDT: specialist multidisciplinary team

Parameter		Traditional referral pathway	CRAM	Total
Response	SMDT review	234 (30.4%)	122 (17.2%)	356 (24.1%)
	Out-patient review	211 (27.4%)	208 (29.3%)	419 (28.3%)
	Local follow up	313 (40.7%)	379 (53.4%)	692 (46.8%)
	More information required	11 (1.4%)	0	11 (0.7%)
Total		769	709	1478

Table 2 shows the referral to first response times in the two groups. The median referral to response time in the CRAM group was five days (IQR 3-6) as compared to eight days (IQR 4-20) in the traditional pathway.

**Table 2:** Referral to first response times in days * Mann-Whitney U test IQR: inter-quartile range; CRAM: clinical radiological assessment meeting

Referral to first response time in days	Traditional referral pathway (days)	CRAM (days)	p-value (*)
Median	8 (IQR 4-20)	5 (IQR 3-6)	p <0.001

## Discussion

There is evidence to suggest that MDT-led cancer care improves diagnostic and staging accuracy and promotes adherence to treatment guidelines; some studies have also reported improvement in oncological outcomes [2, 3, 10]. However, there have been no randomized trials on MDT versus non-MDT cancer care and the benefits are mainly intuitive. Some patients may not benefit due to undue delays and sub-optimal alterations in management, for instance, by a decision to utilize treatment such as pre-operative radiotherapy when surgery alone might be optimal. MDT meetings may also be an important tool for education and training and may help to maintain accurate data for audit and research purposes. 

An emerging problem with many MDTs is an increasing workload with inadequate capacity for a comprehensive presentation of clinical and radiological details, MDT review, discussion, and decision-making. Inclusion of all new referrals, assessment of outcomes on patients undergoing surgery or other treatment modalities and management of patients with recurrent disease limits availability for complex case discussion [11]. For this reason, several surveys have been performed with recommendations aiming to improve MDT efficiency [11-13]. 

The NHS England strategy of ‘MDT Streamlining’ involves classifying the cases listed for MDT into two groups, one group with cases which need time for a detailed discussion due to their complexity, and the other group with more straightforward cases where decisions can be made by following set protocols of standard of care. The prerequisite for the correct classification of the cases into these two groups is to have well-defined standard-of-care protocols developed specific to the type of cancers being discussed in a particular MDT [5].

 One general agreement is that globally, most MDTs struggle with time and resources to provide timely and accurate management plans for cancer care. This is exacerbated in the management of metastatic disease where multiple MDTs may be involved, often remote from the primary hospital MDT, and the patient’s abode. 

Colorectal peritoneal metastases are increasingly being diagnosed at primary staging (synchronous) or follow-up imaging after primary surgical resection of colorectal cancer. Increasing awareness of patients and their relatives by specialist cancer teams, such as medical oncologists, on the potential role of CRS and HIPEC has resulted in a global increase in CPM referrals. Figure 2 illustrates the referral pattern of the past decade with a continuing upward trajectory. These trends are similar in many cancer MDTs.

We instituted a clinical radiological assessment meeting (CRAM) in 2017 to try and address the increasing new referral burden where the primary question is almost always a surgical one 'Will this patient benefit from cytoreductive surgery and hyperthermic intra-peritoneal chemotherapy?'

We have reported a more rapid referral to the first response time and a more manageable peritoneal malignancy specialist MDT. We have incorporated the CRAM into the referral processing pathway and have had positive qualitative feedback from patients, relatives, and referrers.

We believe that a significant benefit of the CRAM 'mini-MDT' is the ability for a surgeon and radiologist to focus together on a new referral, with a surgical and radiological focus on historical and recent imaging. The CRAM improves timely decision-making and increases shared knowledge due to the informality and reduced time pressure of this meeting. Furthermore, the involvement of the administrative team and clinical nurse specialist enhances communication with patients and referrers and promotes team learning.

There are limitations in this study, particularly around retrospective analysis. A criticism of the CRAM might be that we have looked at a soft end-point of referral to first response time as a quality marker with no assessment of other outcome measures such as governance of MDT decisions, adherence to guidelines, patient involvement in decision-making and communication etc [14-17]. We are aware of these limitations but had to explore a pragmatic and practical solution in the face of ever-increasing numbers of referrals, an expanding surgical intervention service, and limited time and resources in a weekly SMDT. 

This study is one of the few evaluations of alternatives to a formal MDT meeting as a mechanism to improve referral processing and MDT efficiency. Similar, or alternative, models may be of use to other cancer MDTs where demands exceed capacity. With advances in machine learning algorithms, Artificial Intelligence tools may be tested in the future. Rigidly following an MDT structure in the face of increasing demand is likely to be counterproductive for patient care, and outcome, and for professionals involved in their care. The CRAM is a cost-effective alternative 'mini-MDT' that facilitates rapid decision-making and allows MDTs to focus on complex cases.

## Conclusions

This study is one of the few evaluations of alternatives to a formal MDT meeting as a mechanism to improve referral processing and MDT efficiency. Similar, or alternative, models may be of use to other cancer MDTs where demands exceed capacity. Rigidly following an MDT structure in the face of increasing demand is likely to be counterproductive for patient care and outcomes as well as for professionals involved in their care. The CRAM is a cost-effective alternative 'mini-MDT' that facilitates rapid decision-making and allows MDTs to focus on complex cases.
